# Whole Genome Sequences of 23 Species from the *Drosophila montium* Species Group (Diptera: Drosophilidae): A Resource for Testing Evolutionary Hypotheses

**DOI:** 10.1534/g3.119.400959

**Published:** 2020-03-27

**Authors:** Michael J. Bronski, Ciera C. Martinez, Holli A. Weld, Michael B. Eisen

**Affiliations:** * Department of Molecular and Cell Biology, University of California, Berkeley; † Berkeley Institute for Data Science, University of California, Berkeley, Berkeley; ‡ Howard Hughes Medical Institute, University of California, Berkeley, Berkeley; § Department of Integrative Biology, University of California, Berkeley, Berkeley

**Keywords:** *Drosophila*, *montium*, genome, assembly

## Abstract

Large groups of species with well-defined phylogenies are excellent systems for testing evolutionary hypotheses. In this paper, we describe the creation of a comparative genomic resource consisting of 23 genomes from the species-rich *Drosophila montium* species group, 22 of which are presented here for the first time. The *montium* group is well-positioned for clade genomics. Within the *montium* clade, evolutionary distances are such that large numbers of sequences can be accurately aligned while also recovering strong signals of divergence; and the distance between the *montium* group and *D. melanogaster* is short enough so that orthologous sequence can be readily identified. All genomes were assembled from a single, small-insert library using MaSuRCA, before going through an extensive post-assembly pipeline. Estimated genome sizes within the *montium* group range from 155 Mb to 223 Mb (mean = 196 Mb). The absence of long-distance information during the assembly process resulted in fragmented assemblies, with the scaffold NG50s varying widely based on repeat content and sample heterozygosity (min = 18 kb, max = 390 kb, mean = 74 kb). The total scaffold length for most assemblies is also shorter than the estimated genome size, typically by 5–15%. However, subsequent analysis showed that our assemblies are highly complete. Despite large differences in contiguity, all assemblies contain at least 96% of known single-copy Dipteran genes (BUSCOs, n = 2,799). Similarly, by aligning our assemblies to the *D. melanogaster* genome and remapping coordinates for a large set of transcriptional enhancers (n = 3,457), we showed that each *montium* assembly contains orthologs for at least 91% of *D. melanogaster* enhancers. Importantly, the genic and enhancer contents of our assemblies are comparable to that of far more contiguous *Drosophila* assemblies. The alignment of our own *D. serrata* assembly to a previously published PacBio *D. serrata* assembly also showed that our longest scaffolds (up to 1 Mb) are free of large-scale misassemblies. Our genome assemblies are a valuable resource that can be used to further resolve the *montium* group phylogeny; study the evolution of protein-coding genes and *cis*-regulatory sequences; and determine the genetic basis of ecological and behavioral adaptations.

Large groups of closely related species with well-defined phylogenetic relationships are invaluable resources with which to investigate evolutionary processes (Drosophila 12 Genomes Consortium *et al.* 2007; Rogers 2018). Previous comparative genomic studies in *Drosophila* have included twelve species spanning the entire *Drosophila* lineage (Drosophila 12 Genomes Consortium *et al.* 2007). Taking into account the short generation time of *Drosophila*, the evolutionary divergence of this sample size space exceeds that of the entire mammalian radiation (Drosophila 12 Genomes Consortium *et al.* 2007; Stark *et al.* 2007). Subsequent sequencing efforts added eight genomes at intermediate evolutionary distances from *D. melanogaster* (Chen *et al.* 2014). While these data sets have provided extraordinary insight into *Drosophila* evolution, they also pose unique challenges. As phylogenetic distance from *D. melanogaster* increases, it becomes more difficult to identify orthologous sequence (Chen *et al.* 2014); and multi-species alignments with divergent sequences can be sensitive to alignment error, especially for small features such as transcription factor binding sites (Stark *et al.* 2007; Kim *et al.* 2009). Accordingly, a data set is needed where 1) distances from *D. melanogaster* are short enough so that orthologous sequence can be readily identified, and 2) species are closely related enough such that sequence similarity produces accurate alignments, but distantly related enough to recover a strong signal of sequence divergence. In this paper, we describe the creation of such a resource by assembling 23 genomes from the *Drosophila montium* species group, which is well-positioned for clade genomics (Rogers 2018). Genomes for 22 of these species are presented here for the first time.

The *montium* clade was initially described as a subgroup within the *melanogaster* species group (Diptera: Drosophilidae) (Hsu 1949; Patterson and Stone 1952; Bock and Wheeler 1972; Bock 1980; Lemeunier *et al.* 1986; Toda 1991), but given its size, Da LageDa Lage *et al.* (2007) later called for its elevation to the rank of species group. While the species group classification was adopted by Yassin (2013, 2018), it has not been used by all authors, and there are advantages and disadvantages to doing so (reviewed in Kopp *et al.* 2019). This species-rich and phenotypically diverse clade contains 94 species (Toda 2019) currently divided into seven subgroups (Yassin 2018). The *montium* group diverged from *D. melanogaster* roughly 28 million years ago (mya) (Russo *et al.* 2013), and the most recent common ancestor of all *montium* species may have lived approximately 19 mya (Yassin 2018). Members of the *montium* group are distributed across Africa, South Asia, South-East Asia, East Asia, and Oceania (Yassin 2018). More than 40 species are currently available in culture, and the list continues to grow. Species from the *montium* group have been used to study a variety of evolutionary, ecological, and behavioral questions, including the genetic basis of female-limited color polymorphism (Yassin *et al.* 2016), cold and desiccation resistance (Kellermann *et al.* 2009), adaptation to drought stress (Ramniwas and Kajla 2012), and courtship behavior (Chen *et al.* 2013, 2019). Previous phylogenetic reconstructions of the *montium* group - typically based on small numbers of genes - have produced incongruent trees, although recent reconstructions have been better resolved (Zhang *et al.* 2003; Da Lage *et al.* 2007; Miyake and Watada 2007; Yang *et al.* 2012; Chen *et al.* 2013, 2019; Yassin *et al.* 2016).

Two *montium* genomes have already been assembled. The *D. kikkawai* genome (Chen *et al.* 2014) was sequenced to a depth of 182x coverage using a combination of 454 and Illumina technology. This produced a 164 Mb assembly with a scaffold N50 of 904 kb. The *D. serrata* genome (Allen *et al.* 2017) was sequenced to a depth of 63x coverage using PacBio long-reads. It yielded a 198 Mb assembly with a contig N50 of 943 kb. While these approaches generated high-quality draft assemblies, the associated costs preclude sequencing dozens of *montium* species this way.

Our goal therefore was to assemble dozens of *montium* genomes in a cost-effective way, while also producing assemblies of sufficient quality and completeness to study protein-coding genes and non-coding sequences genome-wide. In this paper, we describe the sequencing and assembly of 23 *montium* genomes. While our assemblies are relatively fragmented, our analysis shows they are also highly complete. All assemblies contain high percentages of known genes and transcriptional enhancers, and by these measures, they are indistinguishable from far more contiguous *Drosophila* assemblies. Going forward, our assemblies will be a valuable resource that can be used to further resolve the *montium* group phylogeny; study the evolution of protein-coding genes and *cis*-regulatory sequences; and determine the genetic basis of ecological and behavioral adaptations.

## Materials and Methods

### Fly lines

Fly lines for each *montium* species listed in Table 2 were gifts of Artyom Kopp and Michael Turelli, or were acquired from the *Drosophila* Species Stock Center. Additional strain information can be found in the associated BioSample record maintained by NCBI (see Data Availability below).

All fly lines were maintained in small population vials. Prior to sequencing, some lines went through several generations of inbreeding. Other lines were not inbred, either due to difficulty maintaining the fly line, or time limitations.

### Library preparation and sequencing

For each species, DNA was extracted from three female flies using the QIAGEN QIAamp DNA Micro Kit. Sequencing libraries were constructed using the Illumina TruSeq DNA PCR-Free Kit for 350 bp inserts, and visualized on Agilent High Sensitivity DNA chips. Libraries were clustered on Illumina HiSeq 2000 or HiSeq 2500 Systems, generating 100 bp paired-end reads. All sequencing was done at the Vincent J. Coates Genomics Sequencing Laboratory at UC Berkeley. Multiple species were pooled on each lane in an effort to reach sequencing depths of at least 30x per species, assuming genome sizes around 164 Mb (based on the previously published *D. kikkawai* genome (Chen *et al.* 2014)).

### Read exploration and pre-processing

Prior to assembly, read quality and genome / sample characteristics (*e.g.*, estimated genome size, repeat content, and heterozygosity) were explored using FastQC (v. 0.11.2) (Andrews 2010) and String Graph Assembler (SGA) Preqc (v. 0.10.15) (Simpson 2014). SGA Preqc was run using the following commands: sga preprocess, with the option --pe-mode 1; sga index, with the options -a ropebwt and --no-reverse; and sga preqc. The report was generated using the included script sga-preqc-report.py. SGA Preqc estimates genome size using the *k*-mer frequency spectrum of the unassembled reads; and estimates repeat content and heterozygosity using the frequency of repeat and variant branches in a de Bruijn graph, respectively. These measures of repeat content and heterozygosity were used in all subsequent analyses.

Reads from some sequencing runs contained an extra base (*i.e.*, 101 bases instead of 100). This extra base was trimmed using BBDuk (BBMap v. 36.11) (Bushnell), with the option ftr = 99. Reads were adapter-trimmed for known Illumina adapters using BBDuk, with the options ktrim = r, k = 23, mink = 9, hdist = 1, minlength = 75, tpe = t, and tbo = t. The adapter-trimmed reads were then quality-trimmed to Q10 using BBDuk (which implements the Phred algorithm), with the options qtrim = rl, trimq = 10, and minlength = 51.

The *D. cf. bakoue* library was sequenced across two lanes. Sequence quality on the first lane was adversely affected by problematic tiles, as evidenced by the Per Tile Sequence Quality plot generated by FastQC (Andrews 2010). Low-quality reads were removed using FilterByTile (BBMap v. 37.56) (Bushnell), using a statistical profile that included other libraries on the same flowcell. To lower the total sequencing coverage from approximately 75x to 60x, filtered reads from the first lane were subsampled using Reformat (BBMap v. 36.11), with the option samplerate = 0.6.

### Read decontamination

Sequence contaminants were identified by reviewing the Per Sequence GC Content plots from FastQC (Andrews 2010), and the GC Bias plots from SGA Preqc (Simpson 2014). Contaminants formed secondary peaks or spikes in the Per Sequence GC Content plots, and secondary GC % - *k*-mer coverage clusters in the GC Bias plots.

The *D. pectinifera* and *D. vulcana* sequencing libraries were heavily contaminated with microorganisms (mostly bacteria). Low levels of bacteria were also present in the *D. burlai* library. We utilized two different decontamination strategies.

For *D. pectinifera*, we adopted a decontamination strategy similar to Kumar *et al.* (2013). The reads were first assembled using SOAPdenovo2 (Luo *et al.* 2012), with the options -K 49 and -R. Assembled scaffolds at least 1 kb in length were used to create a GC % *vs.* average *k*-mer coverage plot. Scaffolds with 35 <= GC % <= 66 and 40.5 <= average *k*-mer coverage <= 68 were classified as candidate contaminant scaffolds. To avoid removing *Drosophila* scaffolds, candidate contaminant scaffolds were aligned to sequences in NCBI’s Nucleotide database (NCBI Resource Coordinators 2018) using BLASTn (v. 2.2.31+) (Camacho *et al.* 2009). Candidate contaminant scaffolds that aligned to known microorganism sequences were used to create a contaminant reference. Finally, the original reads were aligned to the contaminant reference using Bowtie 2 (v. 2.2.3) (Langmead and Salzberg 2012), with the option --local, and pairs of reads that aligned concordantly were removed prior to the subsequent assembly.

For *D. vulcana* and *D. burlai*, 10,000 reads were sampled from the R1 FASTQ files using seqtk sample (v. 1.0-r75-dirty) (Li 2013), and then converted to FASTA format using seqtk seq. After reviewing the Per Sequence GC Content plot from FastQC (Andrews 2010), potential sequence contaminants were isolated based on their GC %, and then aligned to sequences in NCBI’s Nucleotide database (NCBI Resource Coordinators 2018) using BLASTn (Camacho *et al.* 2009). This led to the identification of closely related bacteria and yeast genomes, which were combined into a contaminant reference. Finally, the original reads were aligned to the contaminant reference using Bowtie 2 (Langmead and Salzberg 2012), with the option --local, and pairs of reads that aligned concordantly were removed prior to assembly.

The *D. burlai*, *D. jambulina*, *D. mayri*, *D. seguyi*, and *D. vulcana* libraries appeared to be contaminated with highly abundant individual sequences, or groups of similar sequences. These sequences created spikes in the Per Sequence GC Content plots from FastQC (Andrews 2010), and corresponded to eight-bp or ten-bp simple sequence repeats (SSRs) that were present in both the forward and reverse reads of the same DNA fragment. The origin of the sequences was unclear. Once the potential contaminant sequences were identified, matching sequences were removed from the reads using BBDuk (Bushnell), with the options k = 75 and hdist = 1.

### Genome GC %

The GC % for each species was calculated using the unassembled reads. Given that the assemblies are depleted of large repeat copies, we thought this approach would produce more accurate estimates than simply calculating the GC % of the assemblies. That being said, raw sequencing data can also have GC biases. Base frequency and read length histograms were constructed using the adapter-trimmed and decontaminated R1 FASTQ files and BBDuk (Bushnell), with the options bhist, lhist, and gcbins = auto. The output was then used to calculate the GC % of the reads, which are reported in Table 2. On average, the GC % of the unassembled reads is 1.5% lower than the GC % of the assemblies (data not shown).

### Choosing an assembler

Exploration of the data using SGA Preqc (Simpson 2014) showed that the *montium* genomes / samples represent a diversity of genome size estimates, repeat contents, heterozygosity levels, and sequencing error rates. Extensive tests were conducted to identify the assembler that performed the best across these diverse samples.

We tested the following assemblers: ABySS (Simpson *et al.* 2009), MaSuRCA (Zimin *et al.* 2013), Meraculous-2D (Goltsman *et al.* 2017 preprint), SOAPdenovo2 (Luo *et al.* 2012), SPAdes (Bankevich *et al.* 2012) / dipSPAdes (Safonova *et al.* 2015), and Velvet (Zerbino and Birney 2008). The resulting assemblies were evaluated using a number of metrics, including contiguity statistics, REAPR (Hunt *et al.* 2013), Feature Response Curves (*FRC*^*bam*^) (Vezzi *et al.* 2012), BUSCO assessments (Simão *et al.* 2015; Waterhouse *et al.* 2018), and the scrutiny of individual enhancer sequences.

### Primary assemblies

All genomes were assembled using MaSuRCA (v. 3.2.2) (Zimin *et al.* 2013), on a server with 48 Intel Xeon E5-2697 v2 2.70 GHz processors and 377 GB of RAM. Assemblies could use up to 36 CPUs. MaSuRCA was supplied with reads that had been force-trimmed to 100 bp and decontaminated, but not adapter-trimmed or quality-trimmed. The authors of MaSuRCA recommend no read trimming, and MaSuRCA performs error correction internally using QuorUM (Marçais *et al.* 2015).

The configuration file for each species contained the insert-size mean and standard deviation for the corresponding sequencing library, as well as the following parameters: GRAPH_KMER_SIZE = auto, USE_LINKING_MATES = 1, CA_PARAMETERS = cgwErrorRate = 0.15, KMER_COUNT_THRESHOLD = 1, and SOAP_ASSEMBLY = 0. The Jellyfish hash size (JF_SIZE) was set to the product of the estimated genome size and coverage.

### Post-assembly pipeline

Our post-assembly pipeline started with assemblies in the MaSuRCA (Zimin *et al.* 2013) output directory 9-terminator, so we could control the gap closing process.

For most assemblies, MaSuRCA (Zimin *et al.* 2013) created several scaffolds with massive gaps (up to 188 kb in length). Given the insert-sizes of the sequencing libraries (∼350 bp), such gap sizes had to be erroneous. Therefore, scaffolds were split on any gap that was unreasonably large relative to the insert-size of the library, and any remaining contigs were retained. Maximum allowed gap sizes were typically around 200–600 bp, depending on the library.

REAPR - Recognition of Errors in Assemblies using Paired Reads (v. 1.0.18) (Hunt *et al.* 2013) was used to identify errors in the assemblies, and to generate new “broken” assemblies that were split on errors occurring over gaps. Errors within contigs were hard-masked with Ns. The command reapr smaltmap was used to align adapter-trimmed reads to the assemblies, and reapr pipeline generated the broken assemblies. Sequences starting with “REAPR_bin” (*i.e.*, the original unmasked sequence) were later filtered from the broken assemblies.

Gaps in the assemblies were closed using a two-step process with adapter-trimmed and quality-trimmed reads. The first round of gap closing was performed using GapCloser (v. 1.12) (Luo *et al.* 2012). This also helped to identify tandem alleles (a type of misassembly) (Pop *et al.* 2004), which GapCloser left as single-N gaps. The second round was done using Sealer (abyss-sealer v. 2.0.2) (Paulino *et al.* 2015), with the option -P 10. For each assembly, “*k* sweeps” typically ranged from *k* = 80 to *k* = 30 (in decrements of 10), but varied if Sealer became stuck on a given *k*-mer size. After two rounds of gap closing, the *D. triauraria* assembly contained more than 2,000 single-N gaps. The remaining single-N gaps and associated flanking sequence were hard-masked with 300 Ns, and Sealer was run a second time using the above settings. This potentially extended the flanking sequence extracted by Sealer beyond the boundaries of the original tandem allele, thereby making it possible to find a connecting path in the graph. This decreased the number of single-N gaps below 2,000.

The assemblies were further improved using Pilon (v. 1.22) (Walker *et al.* 2014), an automated variant detection and genome assembly improvement tool. Adapter-trimmed reads were first aligned to the assemblies using Bowtie 2 (Langmead and Salzberg 2012), with the option --very-sensitive-local. Pilon was then run with the options --fix all,amb, --diploid, and --mingap 1. This attempted to fix SNPs, indels, local misassemblies, and ambiguous bases, as well as fill remaining gaps.

After running Pilon (Walker *et al.* 2014), adapter-trimmed reads were aligned to the improved assemblies using Bowtie 2 (Langmead and Salzberg 2012), with the option --very-sensitive-local. Detailed inspection of the aligned reads showed that many scaffolds were mosaics of multiple haplotypes present in the original samples. This was a significant problem for highly heterozygous samples, as it created numerous recombinant haplotypes not present in the original samples. Our goal therefore was to create “phased” assemblies that reflected the majority haplotype at each variable locus.

Pilon (Walker *et al.* 2014) was run a second time on the improved assemblies, but this time it was used as a variant detection tool to generate VCF files (option --vcf). For highly heterozygous samples, multiple overlapping variants were sometimes present at the same locus, which often led to aberrant phasing behavior. Variants can overlap because they share the same start position, or a large deletion might overlap SNPs or smaller indels. The VCF files were filtered so that only one overlapping variant was retained: either the structural variant (if one was present), or the majority variant. Variants in the VCF files were phased using the read-based phasing tool WhatsHap (v. 0.14.1) (Martin *et al.* 2016 preprint), with the options phase, --ignore-read-groups, --tag = PS, and --indels. BCFtools (v. 1.5) (Li *et al.* 2009) with the options view, --phased or --exclude-phased was then used to create VCF files with only phased or un-phased variants. To facilitate parsing of the phased VCF files, a sequence dictionary was first created with the tool CreateSequenceDictionary from Picard (v. 2.12.1-SNAPSHOT) (Picard), and then VariantsToTable from the Genome Analysis Toolkit (GATK) (v. nightly-2017-09-13-g315c945) (McKenna *et al.* 2010) was used to create tab-delimited tables of variants. For each phase set in the table, the majority haplotype was determined based on the cumulative read count of variants on each haplotype (A or B), with indels weighted half as much as SNPs (because of alignment issues with indels). Phased variants that were present on majority haplotypes were retained. For un-phased variants, the majority allele was retained. New VCF files were then created using only the retained phased and un-phased variants. Finally, BCFtools consensus was used to create new “phased” assemblies by applying the variants in these VCF files to the original “un-phased” assemblies.

Lastly, any remaining ambiguous bases (except N) were randomly assigned to a single base, and scaffolds shorter than 1 kb in length were removed.

### Assembly decontamination

Contaminants in the final assemblies were identified by NCBI’s Contaminant Screen. Most assemblies contained small numbers of scaffolds from bacterial or yeast species, which were removed. A total of four scaffolds across all assemblies also contained suspected adapter / primer sequences. These scaffolds were trimmed if the potential contaminant was located at the end of the scaffold, or split if it was located in the middle of the scaffold.

### Assembly statistics

The correlation between the estimated genome size and the log_10_(repeat content) was calculated using the R (v. 3.4.1) (R Core Team 2017) function cor.test(), with the option method=“pearson”. Repeat content is the frequency of repeat branches in the de Bruijn graph (*k* = 41), as calculated by SGA Preqc (Simpson 2014).

Regression models for predictors of scaffold NG50, and the percentage of the estimated genome size that was assembled, were constructed using the R (R Core Team 2017) function lm(). Heterozygosity and repeat content are the frequency of variant and repeat branches in the de Bruijn graph (*k* = 41), respectively. These values were calculated by SGA Preqc (Simpson 2014).

### Percentage of assembly present in gene-sized scaffolds

**Figure 1:**
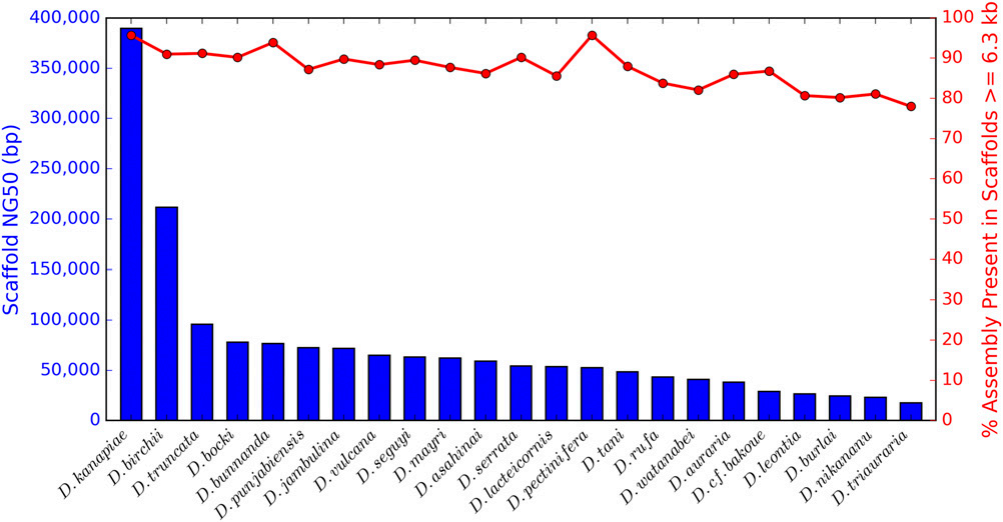
For all *montium* species, the vast majority of the assembly is present in at least gene-sized scaffolds, despite large differences in contiguity. Based on annotations of the previously assembled *D. serrata* genome (NCBI Drosophila serrata Annotation Release 100; Allen *et al.* 2017), the average gene length is up to 6.3 kb. For each *montium* species, the blue bar graph shows the scaffold NG50, and the red line graph shows the percentage of the assembly (total scaffold length) present in scaffolds that are at least 6.3 kb in length. Species are listed in decreasing order of the scaffold NG50.

Figure 1 style adapted from figure in Bradnam *et al.* (2013). The correlation between the log_10_(scaffold NG50) and the percentage of the assembly present in scaffolds greater than or equal to 6.3 kb in length was calculated using the R (R Core Team 2017) function cor.test(), with the option method=“pearson”.

### BUSCO assessment

Eight *montium* assemblies (*D. bocki*, *D. burlai*, *D. jambulina*, *D. kanapiae*, *D. mayri*, *D. pectinifera*, *D. rufa*, and *D. triauraria*) and the *D. melanogaster* reference genome (NCBI Assembly ID: 202931, Release 6 plus ISO1 MT / UCSC Genome Browser Assembly ID: dm6) (Adams *et al.* 2000; Celniker *et al.* 2002; Hoskins *et al.* 2015) were searched for known genes using BUSCO (v. 3.0.2) (Simão *et al.* 2015; Waterhouse *et al.* 2018), with the profile library diptera_odb9. The configuration file included the option mode = genome, along with the following default settings: evalue = 1e-3, limit = 3, and long = False. The BUSCO plot was constructed using the included script generate_plot.py.

BUSCO ID: EOG09150BMT is the gene that appears to be completely missing across all eight *montium* species. According to OrthoDB (Kriventseva *et al.* 2019), the *D. melanogaster* ortholog is *CG14965*, which encodes a THAP domain transcription factor (Roussigne *et al.* 2003; Hammonds *et al.* 2013; Thurmond *et al.* 2019).

### Whole genome alignment pipeline

Each *montium* genome was individually aligned to the *D. melanogaster* genome (NCBI Assembly ID: 202931, Release 6 plus ISO1 MT / UCSC Genome Browser Assembly ID: dm6) (Adams *et al.* 2000; Celniker *et al.* 2002; Hoskins *et al.* 2015) using a previously described whole genome alignment pipeline (UCSC Genome Bioinformatics Group n.d.-ab; Kent *et al.* 2003). Target and query genomes were repeat-masked using RepeatMasker (v. open-4.0.7) (Smit *et al.* 2013–2015), with the sequence search engine RMBlast (v. 2.2.28) and Tandem Repeat Finder (TRF) (v. 4.04) (Benson 1999), and the options -s, -species drosophila, -gccalc, -nocut, and -xsmall. Repeats were soft-masked by converting the corresponding sequence to lowercase. Pairs of genomes were aligned using LASTZ (v. 1.04.00) (Harris 2007), with the following options from Chen *et al.* (2014): target_genome[multiple], --masking = 50, --hspthresh = 2200, --ydrop = 3400, --gappedthresh = 4000, --inner = 2000, and --format = axt. Repeats were masked in the previous step so that lowercase sequence could be ignored during LASTZ’s seeding stage (Harris 2007). The LASTZ alignments were then processed into structures called “chains” and “nets” (UCSC Genome Bioinformatics Group n.d.-c) using a series of programs described in detail by Kent *et al.* (2003). Briefly, FASTA files for the target and query assemblies were converted to 2bit format using faToTwoBit. Files containing chromosome / scaffold lengths were created using faSize with the option -detailed. Gapless alignments (“blocks”) were linked together into maximally scoring chained alignments, or chains. The order of blocks within chains must be the same in both target and query genomes. Blocks within chains can be separated by insertions / deletions, inversions, duplications, or translocations. Chains were built using axtChain with the option -linearGap = medium, and then filtered using chainPreNet. Gaps in high-scoring chains were filled in with lower scoring chains, creating hierarchies (parent-child relationships) known as nets. Nets were constructed using chainNet with the option -minSpace = 1, and then annotated using netSyntenic. Finally, subsets of chains found in nets were extracted using netChainSubset, creating liftOver chain files.

### Identification of montium sequences orthologous to D. melanogaster enhancers

Kvon et al. (2014) previously described a large set of DNA fragments (Vienna Tiles) that drive expression in the *D. melanogaster* embryo. These fragments are approximately 2 kb in length. A total of 3,457 fragments were positive for enhancer activity and PCR-verified. *D. melanogaster* coordinates were remapped onto each *montium* assembly using liftOver (Hinrichs *et al.* 2006), with the options -minMatch = 0.1 and -multiple. The liftOver program was originally written to remap coordinates between assemblies of the same species. However, it is routinely used for interspecies lifts, and in our experience, it performed well. In cases of multiple remappings for a single fragment, the larger coordinate span was retained, as it typically contained the sequence of interest.

The following strategy was used to identify reciprocal best hits. The candidate orthologs from each *montium* assembly were aligned back to the *D. melanogaster* genome using BLASTn (Camacho *et al.* 2009), with the options -evalue 0.00029, -word_size 11, -reward 2, -penalty -3, -gapopen 5, -gapextend 2, -dust no, and -outfmt 6. The E value was set to the reciprocal of the number of enhancer sequences: 1 / 3,457 or 0.00029. BEDTools (v. 2.17.0) (Quinlan and Hall 2010) intersect was then used to determine whether the highest scoring BLAST hit for each *montium* sequence overlapped the original fragment coordinates in the *melanogaster* genome. Conversely, the *melanogaster* fragment sequences were aligned to each *montium* assembly using BLASTn, and BEDTools intersect was used to determine whether the highest scoring BLAST hit for each *melanogaster* sequence overlapped the remapped fragment coordinates in the *montium* assembly. Fragments that met both criteria were classified as reciprocal best hits.

To visualize the similarity between reciprocal best hits, pairs of *montium* (subject) and *melanogaster* (query) sequences were aligned using BLASTn (Camacho *et al.* 2009), with the options -task blastn-short, -evalue 0.00029, -reward 2, -dust no, and -outfmt 6. The BLAST output was filtered so that lower-scoring hits nested within, or partially overlapping, higher-scoring hits were removed / trimmed. The resulting hits were used to calculate the query coverage and length-weighted percent identity for the alignment.

### Scaffold alignment visualization using dotplots

We aligned our *D. serrata* assembly (strain 14028-0681.02) to the previously published *D. kikkawai* (Chen *et al.* 2014) and PacBio *D. serrata* (strain Fors4) (Allen *et al.* 2017) assemblies using the whole genome alignment pipeline detailed above (UCSC Genome Bioinformatics Group n.d.-ab; Kent *et al.* 2003).

Pairs of orthologous scaffolds / contigs were aligned for visualization using LASTZ (Harris 2007), with the following options (in part from Chen *et al*. (2014)): --chain, --masking = 50, --hspthresh = 2200, --ydrop = 3400, --gappedthresh = 4000, --inner = 2000, and --format = rdotplot. For consistent visualization, our scaffolds scf7180000628572 and scf7180000629414 were reverse-complemented prior to pairwise alignment. Dotplots were constructed using R (R Core Team 2017).

### Scripting and plotting

Unless otherwise stated, all scripts were written in Python (v. 2.7.14) (van Rossum and de Boer 1991), and plots were created using Matplotlib (v. 1.5.1) (Hunter 2007).

### Data availability

Table S1 reports a regression analysis for predictors of scaffold NG50. Table S2 reports a regression analysis for predictors of the percentage of the estimated genome size that was assembled. Figure S1 contains NG graphs showing the distribution of scaffold lengths for 23 *montium* assemblies. Figure S2 contains additional dotplots.

All assemblies and sequencing data are publicly available through the *Drosophila montium* Species Group Genomes Project, NCBI BioProject Accession PRJNA554346. This record provides links to the assemblies, BioSamples, and sequencing data. The Whole Genome Shotgun projects have been deposited at DDBJ/ENA/GenBank under the accession numbers listed in Table 2. The versions described in this paper are versions XXXX01000000. Raw sequencing data were uploaded to the NCBI Sequence Read Archive (SRA). Besides removing reads that did not pass filtering, the FASTQ files were unprocessed.

Sequencing libraries for *D. cf. bakoue*, *D. kanapiae*, *D. mayri*, *D. punjabiensis*, *D. tani*, *D. truncata*, and *D. vulcana* were spread across two lanes of a flowcell. When the FASTQ files were uploaded to the NCBI SRA, the R1 and R2 files from both lanes were combined into individual R1 and R2 FASTQ files. If users wish to demultiplex reads by lane for these samples, lane information (always 1 or 2) is preserved in the sequence identifier line of the original FASTQ files.

Repeat-masked assemblies, RepeatMasker (Smit *et al.* 2013–2015) annotation / summary tables, liftOver chain files from whole genome alignments, and BUSCO assessment tables were deposited in the Dryad repository: https://doi.org/10.6078/D1CH5R. Supplemental material available at figshare: https://doi.org/10.25387/g3.11301932.

## Results and Discussion

### Genome size estimates and assembly statistics

To assemble dozens of genomes in a cost-effective way, we sequenced a single, small-insert (350 bp), PCR-free, library to roughly 35x coverage for each species. The genomes were assembled using the Maryland Super Read Cabog Assembler (MaSuRCA), which combines de Bruijn graph and overlap-layout-consensus (OLC) approaches into a novel algorithm based on “super-reads” (Zimin *et al.* 2013). The genomes then went through an extensive post-assembly pipeline to further improve the primary assemblies. See the Materials and Methods for an in-depth description of the entire pipeline.

Table 1 reports genome size estimates and assembly statistics (total scaffold length, scaffold / contig NG50, length of longest scaffold / contig, and total gap length) for 23 *montium* species. Genome size estimates are based on the *k*-mer frequency spectrum of the unassembled reads, as calculated by String Graph Assembler (SGA) Preqc (Simpson 2014). The scaffold / contig NG50 (Earl *et al.* 2011; Bradnam *et al.* 2013) is analogous to the well-known N50, but substitutes the estimated genome size for the total assembly length. For example, a scaffold NG50 of 100,000 bp means that 50% of the estimated genome size is present in scaffolds that are at least 100,000 bp. When this calculation is repeated for all integers from 1 to 100, the result is an “NG graph” (Bradnam *et al.* 2013). Figure S1 contains NG graphs showing the distribution of scaffold lengths for each *montium* assembly. Table 2 contains additional sample information, including the strain name, coverage, and GC %. The table also reports the frequency of variant and repeat branches in de Bruijn graphs constructed by SGA Preqc (Simpson 2014), which are estimates of heterozygosity and repeat content, respectively. These measures of heterozygosity and repeat content were used in all subsequent analyses.

**Table 1:** Genome size estimates and assembly statistics

Species	Est. Genome Size (bp)	Total Scaffold Length (bp)	Scaffold NG50 (bp)	Longest Scaffold (bp)	Contig NG50 (bp)	Longest Contig (bp)	Total Gap Length (bp)
*D. kanapiae*	155,490,160	152,203,088	389,587	2,274,126	301,459	2,274,126	205,953
*D. birchii*	169,148,727	156,593,892	211,718	1,501,252	164,580	1,183,551	81,207
*D. truncata*	190,688,284	167,897,087	95,737	830,117	73,889	827,712	375,696
*D. bocki*	155,095,574	151,202,254	78,068	785,450	65,314	785,450	69,231
*D. bunnanda*	181,250,127	151,823,105	76,713	1,142,480	65,403	1,127,760	152,423
*D. punjabiensis*	197,448,094	192,339,030	72,420	1,226,934	64,043	1,083,757	153,372
*D. jambulina*	179,468,675	163,991,206	71,637	873,064	61,348	756,687	61,365
*D. vulcana*	209,187,412	187,578,810	65,096	530,507	51,774	472,464	116,296
*D. seguyi*	206,814,592	178,856,532	63,109	891,413	54,123	891,413	233,653
*D. mayri*	223,398,425	167,807,061	62,249	2,219,437	43,922	1,355,909	364,662
*D. asahinai*	216,977,949	189,050,820	59,266	1,052,132	50,528	904,342	75,577
*D. serrata*	184,673,878	159,679,625	54,224	1,091,401	43,626	718,797	79,019
*D. lacteicornis*	203,475,870	182,681,050	53,799	1,044,495	44,105	766,914	60,537
*D. pectinifera*	220,219,034	149,209,000	52,632	528,734	41,478	467,725	58,142
*D. tani*	194,820,185	180,972,673	48,517	921,527	44,341	780,901	135,927
*D. rufa*	210,769,271	186,167,886	43,287	498,065	38,201	498,065	80,447
*D. watanabei*	182,199,997	196,825,890	40,952	1,045,963	36,818	656,929	135,921
*D. auraria*	220,036,088	197,420,731	38,365	491,046	35,679	491,046	130,216
*D. cf. bakoue*	219,308,053	187,248,584	28,924	1,045,797	27,520	598,596	58,713
*D. leontia*	162,918,854	164,601,511	26,461	331,217	23,897	301,031	91,716
*D. burlai*	198,129,694	175,666,184	24,417	628,960	23,536	628,960	153,793
*D. nikananu*	217,706,973	190,505,469	23,001	626,542	21,180	574,375	226,904
*D. triauraria*	217,036,792	197,369,186	17,513	590,840	16,493	576,941	156,570

Genome size estimates were calculated by SGA Preqc (Simpson 2014) based on the *k*-mer frequency spectrum of the unassembled reads. To calculate the scaffold NG50 (Earl *et al.* 2011; Bradnam *et al.* 2013), scaffold lengths were ordered from longest to shortest and then summed, starting with the longest scaffold. The NG50 was the scaffold length that brought the sum above 50% of the estimated genome size. Contig lengths were estimated by splitting scaffolds on every N, including single Ns. Species are listed in decreasing order of scaffold NG50.

**Table 2:** Additional sample and genome information

Species	Strain	NCBI Accession #	Coverage (x)	GC %	Freq. Variant Branches (*k* = 41)	Freq. Repeat Branches (*k* = 41)
*D. asahinai*	E-12502 (TKNK40)	VNJZ00000000	28	40.74	5.988E-04	4.636E-04
*D. auraria*	14028-0471.01	VNJW00000000	39	40.32	4.143E-04	4.930E-04
*D. cf. bakoue*	São Tomé Light	VNJL00000000	59	41.98	2.850E-03	4.005E-04
*D. birchii*	14028-0521.00	VNKA00000000	36	39.89	1.639E-04	2.991E-04
*D. bocki*	E-12901 (IR2-37)	VNJY00000000	41	40.32	1.961E-04	2.103E-04
*D. bunnanda*	14028-0721.00	VNKE00000000	33	40.01	5.872E-04	2.895E-04
*D. burlai*	14028-0781.00	VNJT00000000	45	40.51	3.317E-03	3.401E-04
*D. jambulina*	14028-0671.01	VNJX00000000	32	40.61	1.230E-03	2.458E-04
*D. kanapiae*	14028-0541.00	VNJM00000000	38	39.80	1.479E-04	1.175E-04
*D. lacteicornis*	E-14104 (ISGB1)	VNKF00000000	25	40.26	NA	NA
*D. leontia*	RGN 210-13	VNKB00000000	37	40.24	3.179E-03	2.686E-04
*D. mayri*	14028-0591.01	VNJN00000000	34	38.40	9.486E-04	6.182E-04
*D. nikananu*	14028-0601.01	VNJV00000000	40	40.36	3.686E-03	5.446E-04
*D. pectinifera*	14028-0731.00	VNKC00000000	22	38.05	NA	NA
*D. punjabiensis*	14028-0531.01 or MYS-170-D	VNJR00000000	47	39.39	2.047E-03	2.967E-04
*D. rufa*	E-14802 (EHO91)	VNKH00000000	29	40.55	3.982E-04	4.826E-04
*D. seguyi*	14028-0671.02	VNJU00000000	38	39.11	1.145E-03	4.656E-04
*D. serrata*	14028-0681.02	VNKD00000000	31	38.61	1.098E-03	3.301E-04
*D. tani*	14020-0011.00	VNJO00000000	40	40.02	1.221E-03	3.405E-04
*D. triauraria*	14028-0691.01	VNKG00000000	28	39.95	3.047E-03	6.126E-04
*D. truncata*	RGN23	VNJQ00000000	51	37.89	2.813E-04	3.785E-04
*D. vulcana*	14028-0711.00	VNJP00000000	29	41.70	2.531E-04	3.151E-04
*D. watanabei*	14028-0531.02	VNJS00000000	38	38.99	3.715E-03	2.936E-04

For *D. punjabiensis*, we sequenced one of two potential strains. Additional sequencing is underway to confirm the strain identification. Coverage is equal to the total amount of sequencing data (after read decontamination) divided by the estimated genome size (from SGA Preqc (Simpson 2014)). The GC % is based on the unassembled reads, not the assembly. See the Materials and Methods for additional information. The frequency of variant and repeat branches in the de Bruijn graph (*k* = 41) was calculated by SGA Preqc. A *k*-mer size of 41 was chosen to maximize the number of species that could be compared. Sequence coverage was too low to estimate these parameters at *k* = 41 for *D. lacteicornis* and *D. pectinifera*.

Estimated genome sizes within the *montium* group range from 155.1 Mb to 223.4 Mb (mean = 196.4 Mb; median = 198.1 Mb). These sizes are consistent with the previously assembled *D. kikkawai* (Chen *et al.* 2014) and *D. serrata* (Allen *et al.* 2017) genomes, with total sequence lengths of 164.3 Mb and 198.0 Mb, respectively. Our own *D. serrata* assembly (strain 14028-0681.02) has an estimated genome size of 184.7 Mb. The relatively small difference between our genome size estimate and the total contig length of the previously published PacBio *D. serrata* assembly (strain Fors4) (Allen *et al.* 2017) is likely a product of the imprecision of *k*-mer frequency spectrum-based genome size estimates, along with strain-level differences in genome size. Across all *montium* species, estimated genome size is strongly positively correlated with repeat content (*r* = 0.88, *p* < 1e-06).

Scaffold NG50s vary widely, from the remarkably contiguous *D. kanapiae* assembly (389,587 bp), to the highly fragmented *D. triauraria* assembly (17,513 bp). The contiguity of the *D. kanapiae* assembly is somewhat surprising, given the use of a small-insert library, but is related to genome and sample characteristics described below. The average scaffold NG50 across all *montium* species is 73,813 bp (median = 54,224 bp).

Multiple factors can influence the contiguity of an assembly, including repeat content, heterozygosity, and sequencing depth. Large, repeat-rich genomes are typically difficult to assemble, as are highly heterozygous samples (Simpson 2014). Given that the *montium* genomes were assembled using small-insert libraries (350 bp), they are especially sensitive to repeat content and heterozygosity. In the absence of long-distance information, in the form of mate-pair libraries or long-reads, large repeats form unresolvable structures in the graph. This results in fragmented assemblies that are missing many repeat copies (Potato Genome Sequencing Consortium *et al.* 2011; Treangen and Salzberg 2011). Similarly, high levels of heterozygosity can create complicated graph structures that cause breaks in the assembly (Pryszcz and Gabaldón 2016; Dominguez Del Angel *et al.* 2018). Assemblers like Meraculous-2D (Goltsman *et al.* 2017 preprint) and Platanus (Kajitani *et al.* 2014) that are designed to handle high levels of heterozygosity typically require mate-pair libraries. Finally, areas of low sequence coverage can also fragment an assembly (Simpson 2014).

Repeat content and heterozygosity vary widely across genomes / samples (Table 2), which in turn drive the scaffold NG50. For example, the *D. kanapiae* assembly owes its impressive contiguity to the lowest repeat content and heterozygosity level of any *montium* species. In contrast, the highly fragmented *D. triauraria* assembly combines the second highest repeat content with the fifth highest level of heterozygosity. To investigate the combined effect of repeat content, heterozygosity, and coverage on the scaffold NG50 across all *montium* species, we constructed a simple regression model (Table S1). As expected, the scaffold NG50 is inversely proportional to repeat content and heterozygosity, with repeat content impacting assemblies nearly twice as much as heterozygosity. The scaffold NG50 is generally unaffected by sequencing depth, as most genomes reach the minimum coverage necessary to effectively assemble contigs. While the sample size is small, the regression results are reassuring in that for each variable, the direction and relative magnitude of change is consistent with general genome assembly predictions.

The total scaffold length for most *montium* assemblies reaches 85–95% of the estimated genome size. In Figure S1, this is where the curves intersect the *x*-axis. Given that our assemblies are missing many large repeat copies (see above), they should generally be shorter than the estimated genome size, with the magnitude of the difference proportional to the number, size, and divergence of repeat copies (Alkan *et al.* 2011). For example, the *D. pectinifera* and *D. mayri* assemblies reach only 67.8% and 75.1% of their estimated genome sizes, respectively. *D. mayri* has the highest repeat content of any *montium* species (Table 2), and *D. pectinifera* has the second largest estimated genome size (Table 1), which is highly correlated with repeat content. (The *D. pectinifera* sample was also heavily contaminated with bacteria. While the bacterial reads were filtered prior to assembly, their initial presence lowered the sequencing coverage of the fly genome. This further shortened the assembly, and prevented SGA Preqc (Simpson 2014) from estimating repeat content and heterozygosity.) In contrast, the relatively small and repeat-poor *D. kanapiae* genome yielded an assembly that reaches 97.9% of its estimated genome size.

Compared to repeat content, heterozygosity can act as an opposing force on the total scaffold length. Given modest levels of heterozygosity, most assemblers collapse allelic variation into a single consensus sequence. As heterozygosity increases though, divergent haplotypes can sometimes be assembled independently on different scaffolds (Pryszcz and Gabaldón 2016; Dominguez Del Angel *et al.* 2018). This artificially inflates the total scaffold length, and closes the gap between the estimated genome size and assembly length. (Some assemblers can also over-assemble the data and produce many small contigs / scaffolds known as “chaff” (Salzberg *et al.* 2012).) Consistent with this effect, the total scaffold lengths for *D. leontia* and *D. watanabei* actually exceed their estimated genome sizes. In Figure S1, these curves never intersect the *x*-axis. In the case of *D. watanabei*, this difference is large: 14.6 Mb. *D. watanabei* has the highest heterozygosity level of any *montium* species (Table 2), while *D. leontia* ranks fourth.

To investigate the combined effect of repeat content, heterozygosity, and coverage on the percentage of the estimated genome size that was assembled across all *montium* species, we constructed a simple regression model (Table S2). As expected, the percentage of the estimated genome size that was assembled is inversely proportional to repeat content, but positively correlated with heterozygosity - with repeat content being the primary driver. Once again, the sample size is small for a regression analysis, and the heterozygosity results only reach statistical significance at an alpha level of 0.10, but the results are generally as expected.

Overall, the *montium* assemblies are fragmented, as evidenced by their modest scaffold NG50s. However, taken in isolation, the NG50s say little about the quality of the assemblies. Any single metric (especially the NG50) can be a poor predictor of the quality / utility of an assembly. It is best to evaluate assemblies using a variety of methods, with an eye toward the downstream application (Bradnam *et al.* 2013). For example, it is often advantageous to sacrifice contiguity for accuracy, and many questions can be answered without knowing the detailed repeat structure of the genome. We turn now to evaluating the *montium* assemblies in ways that will tell us if they are of sufficient contiguity and quality to study genes and transcriptional enhancers genome-wide.

### The vast majority of montium scaffolds are at least gene-sized

To study genes, a genome assembly should be present in at least gene-sized fragments (Yandell and Ence 2012; Bradnam *et al.* 2013). By extension, such an assembly would also be useful for studying any features that are gene-sized or smaller, such as enhancers. Based on existing annotations of the PacBio *D. serrata* genome, the average gene length is up to 6.3 kb (NCBI Drosophila serrata Annotation Release 100; Allen *et al.* 2017). Figure 1 shows the relationship between the scaffold NG50 and the percentage of the assembly (total scaffold length) present in scaffolds that are at least 6.3 kb in length. Most *montium* assemblies are significantly shorter than their estimated genome sizes, on account of missing repeats. Therefore, we think it’s reasonable to ask the question: What percentage of the non-repetitive genome is present in at least gene-sized scaffolds? If we instead used the estimated genome sizes, the percentages would obviously decrease. Despite large differences in contiguity, all assemblies are present predominantly as scaffolds that are at least gene-sized. While there is a clear downward trend with decreasing NG50 (*r* = 0.79, *p* < 1e-5), in practice, this effect is modest. Even for the most fragmented assemblies, roughly 80% of the assembly is present in at least gene-sized fragments.

### All montium assemblies contain high percentages of known genes

**Figure 2:**
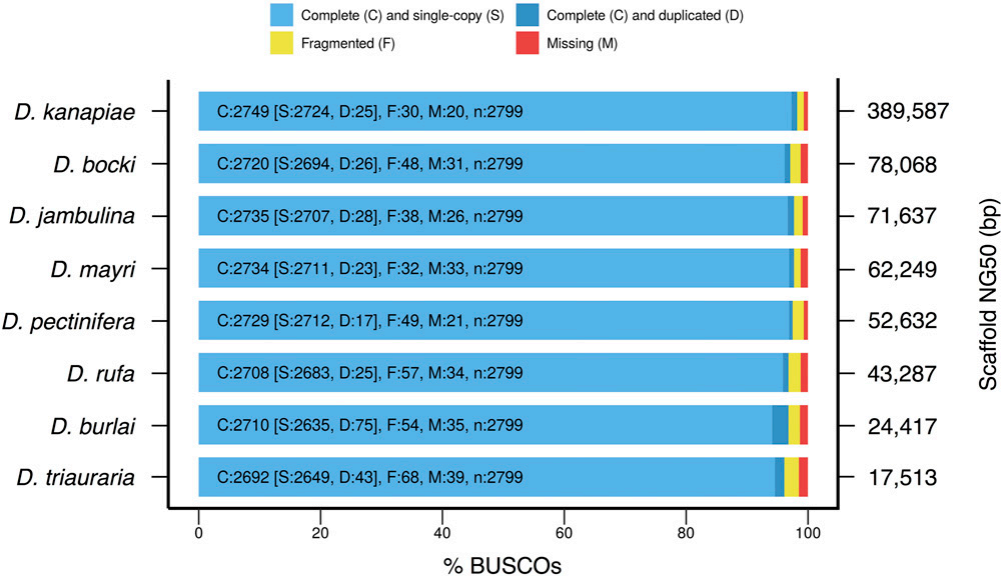
All *montium* assemblies contain high percentages of known genes despite large differences in contiguity. BUSCO (Simão *et al.* 2015; Waterhouse *et al.* 2018) assessment results for eight *montium* genomes representing a diversity of genomes / assemblies. The Dipteran BUSCO set contains 2,799 genes. For each assembly, the bar graph reports the number of BUSCOs that are complete and single-copy, complete and duplicated, fragmented, and missing. The scaffold NG50 for each assembly is shown on the right.

The vast majority of scaffolds in each *montium* assembly are large enough to contain genes. However, do the scaffolds actually contain known genes? One way to assess the quality of an assembly is by annotation completeness: a good assembly should contain a high percentage of known genes. Benchmarking Universal Single-Copy Orthologs (BUSCOs) are single-copy genes present in more than 90% of surveyed species (Simão *et al.* 2015; Waterhouse *et al.* 2018). The Dipteran BUSCO set contains 2,799 genes, and is based on a survey of 25 species. Figure 2 shows the BUSCO assessment results for eight *montium* assemblies. These species were chosen for their diversity: they occupy most subgroups in the *montium* group phylogeny (Yassin 2018); include assemblies that fall far short of their estimated genome size; and represent a diversity of genome size estimates, repeat contents, sample heterozygosity levels, and assembly contiguities. They range from the small, repeat-poor, homozygous, and contiguous *D. kanapiae* (estimated genome size = 155 Mb, scaffold NG50 = 390 kb), to the large, repeat-rich, highly heterozygous, and fragmented *D. triauraria* (estimated genome size = 217 Mb, scaffold NG50 = 18 kb). Strikingly, despite the wide range of contiguities, there is little variation in gene content: at least 96.1% of BUSCOs are complete (single-copy or duplicated) across all species. The *D. kanapiae* assembly exceeds 98%. For comparison, the previously assembled *D. kikkawai* and *D. serrata* genomes, which approach scaffold / contig N50s of 1 Mb, reach 98.1% and 96.2%, respectively (Allen *et al.* 2017). Once again, despite their relatively modest scaffold NG50s, our assemblies have performed well in metrics that matter for downstream analyses.

Ten BUSCOs were classified as missing across all eight *montium* species. Missing BUSCOs can be due to genuine loss events, incomplete assemblies, or incorrect classifications (Simão *et al.* 2015; Waterhouse *et al.* 2018). For comparison, sixteen BUSCOs were classified as missing in the *D. melanogaster* reference assembly (data not shown), including nine of the ten BUSCOs that appear to be missing in the *montium* clade. Given that *D. melanogaster* was used to construct the Dipteran BUSCO set, and contributed orthologs for all 2,799 genes, no BUSCO should in theory be classified as missing in the reference assembly. However, high sequence divergence and complex gene structures can make BUSCOs difficult to identify in an assembly, making it appear as if fragmented or even complete genes are missing (Simão *et al.* 2015; Waterhouse *et al.* 2018). After reviewing the *melanogaster* and *montium* BUSCO output in detail, nine of the ten BUSCOs classified as missing in all eight *montium* species appear to be present in at least fragmented form. Only one BUSCO, a gene encoding a THAP domain transcription factor (Roussigne *et al.* 2003; Hammonds *et al.* 2013; Thurmond *et al.* 2019), appears to be completely missing across the surveyed *montium* species.

### Whole genome alignments of montium species to D. melanogaster

Given that the *montium* assemblies contain high percentages of known genes, we next determined if they also contain large percentages of known transcriptional enhancers. Our motivation for doing so was twofold. First of all, we want to use this data set to study enhancer evolution, so we needed to confirm that such sequences were present in the assemblies. Second, to the extent that non-coding regions can be more challenging to assemble than genic regions, this served as an additional completeness test. To facilitate the identification of enhancer sequences in *montium* genomes, we aligned each *montium* assembly to the *D. melanogaster* genome using a previously described whole genome alignment pipeline (UCSC Genome Bioinformatics Group n.d.-ab; Kent *et al.* 2003). See the Materials and Methods for a complete description. Briefly, each *montium* assembly was individually aligned to the *D. melanogaster* genome using LASTZ (Harris 2007). The LASTZ alignments were then processed into structures called “chains” and “nets” (UCSC Genome Bioinformatics Group n.d.-c) using a series of programs described in detail by Kent *et al.* (2003). The pipeline ultimately produced liftOver chain files. Given a set of coordinates for an annotated feature in the *D. melanogaster* genome, the liftOver (Hinrichs *et al.* 2006) utility returns coordinates for the (putatively) orthologous sequence in an aligned *montium* genome. For this analysis, we also included the previously assembled *D. kikkawai* genome (Chen *et al.* 2014).

### All montium assemblies contain thousands of D. melanogaster enhancer orthologs

**Figure 3:**
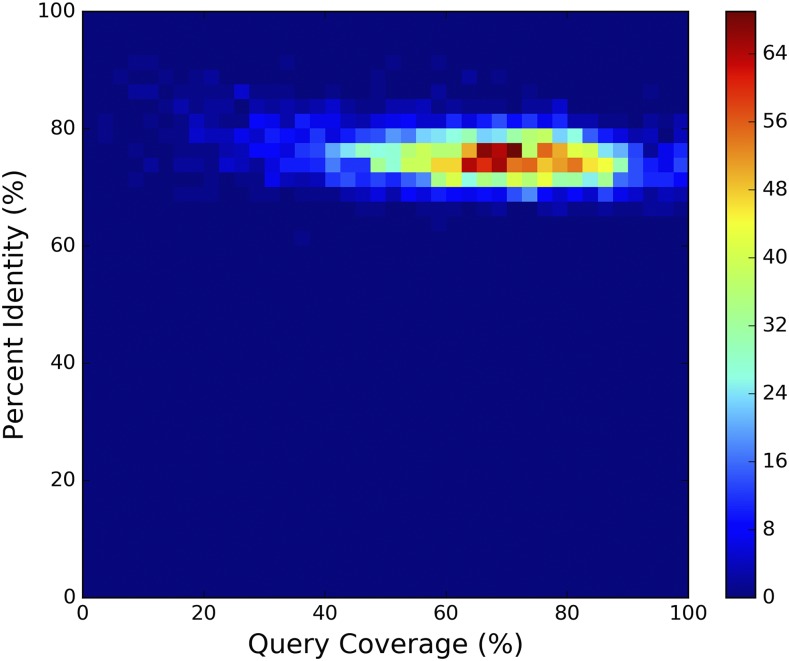
Pairwise BLASTn alignments between *D. melanogaster* enhancers and *D. lacteicornis* orthologs show highly similar sequences. 3,457 experimentally verified *D. melanogaster* enhancers from Kvon *et al.* (2014) were remapped onto the *D. lacteicornis* assembly using liftOver (Hinrichs *et al.* 2006). This yielded 3,375 reciprocal best hits between the *D. melanogaster* and *D. lacteicornis* genomes. *D. lacteicornis* was chosen for illustrative purposes because the assembly is close to the median scaffold NG50. The 2D histogram shows query coverage and percent identity for 3,375 pairwise *D. melanogaster* - *D. lacteicornis* BLASTn (Camacho *et al.* 2009) alignments. Query coverage is the percentage of *D. melanogaster* sequence that aligned to *D. lacteicornis* sequence; and percent identity is the length-weighted percent identity for hits in the alignment.

With the genomes aligned, we turned to looking for known enhancer sequences in the *montium* assemblies. We used a previously described set of 3,500 experimentally verified transcriptional enhancers that drive expression in the *D. melanogaster* embryo (Kvon *et al.* 2014). The enhancer-containing sequences (tiles) were approximately 2 kb in length. Using liftOver (Hinrichs *et al.* 2006), we remapped the *melanogaster* coordinates onto each *montium* assembly. Across all *montium* assemblies, at least 99.6% of enhancer coordinates were successfully remapped (Table 3). To determine whether the remapped coordinates correspond to orthologous sequence, we used BLASTn (Camacho *et al.* 2009) to align the *montium* sequences back to the *melanogaster* genome, and the *melanogaster* sequences to the *montium* genomes. On average, 96.5% of remapped coordinates are reciprocal best hits between the two genomes (Table 3). Of note, the highly contiguous *D. kikkawai* genome (Chen *et al.* 2014) is indistinguishable from our more fragmented assemblies. Next, we aligned each *melanogaster* sequence to its putative *montium* ortholog using BLASTn. Figure 3 shows illustrative results for the *D. lacteicornis* assembly, which is close to the median scaffold NG50. On average, 65.3% of the *D. melanogaster* sequence aligns to sequence from *D. lacteicornis* (query coverage). The average percent identity is 75.1%, and the Expect value (E) for the vast majority of alignments is essentially zero (data not shown). Based on these results, it is clear that we can remap coordinates for thousands of *D. melanogaster* enhancers onto any *montium* assembly, and with a high level of confidence extract orthologous sequences.

**Table 3:** Thousands of orthologous* montium* enhancers can be identified by remapping *D. melanogaster* enhancer coordinates onto *montium* assemblies

Species	Attempted Remappings	Successful Remappings	% Successful Remappings	Reciprocal Best Hits (RBH)	% Successful Remappings that are RBH
*D. asahinai*	3,457	3,450	99.8	3,361	97.4
*D. auraria*	3,457	3,448	99.7	3,347	97.1
*D. cf. bakoue*	3,457	3,451	99.8	3,275	94.9
*D. birchii*	3,457	3,449	99.8	3,385	98.1
*D. bocki*	3,457	3,449	99.8	3,377	97.9
*D. bunnanda*	3,457	3,447	99.7	3,359	97.4
*D. burlai*	3,457	3,450	99.8	3,272	94.8
*D. jambulina*	3,457	3,450	99.8	3,327	96.4
*D. kanapiae*	3,457	3,449	99.8	3,406	98.8
*D. kikkawai*	3,457	3,449	99.8	3,377	97.9
*D. lacteicornis*	3,457	3,451	99.8	3,375	97.8
*D. leontia*	3,457	3,444	99.6	3,247	94.3
*D. mayri*	3,457	3,449	99.8	3,384	98.1
*D. nikananu*	3,457	3,451	99.8	3,221	93.3
*D. pectinifera*	3,457	3,449	99.8	3,383	98.1
*D. punjabiensis*	3,457	3,449	99.8	3,266	94.7
*D. rufa*	3,457	3,452	99.9	3,368	97.6
*D. seguyi*	3,457	3,444	99.6	3,334	96.8
*D. serrata*	3,457	3,447	99.7	3,350	97.2
*D. tani*	3,457	3,451	99.8	3,301	95.7
*D. triauraria*	3,457	3,448	99.7	3,258	94.5
*D. truncata*	3,457	3,449	99.8	3,366	97.6
*D. vulcana*	3,457	3,448	99.7	3,358	97.4
*D. watanabei*	3,457	3,449	99.8	3,147	91.2

Coordinates for *D. melanogaster* enhancers from Kvon *et al.* (2014) were remapped onto aligned *montium* assemblies using liftOver (Hinrichs *et al.* 2006). Reciprocal best hits (RBH) were identified by aligning *montium* sequences back to the *melanogaster* genome, and *melanogaster* sequences to the *montium* genomes - both using BLASTn (Camacho *et al.* 2009). See Materials and Methods for additional details. For comparison, we also included the previously assembled *D. kikkawai* genome (Chen *et al.* 2014).

### Identifying potential misassemblies

**Figure 4:**
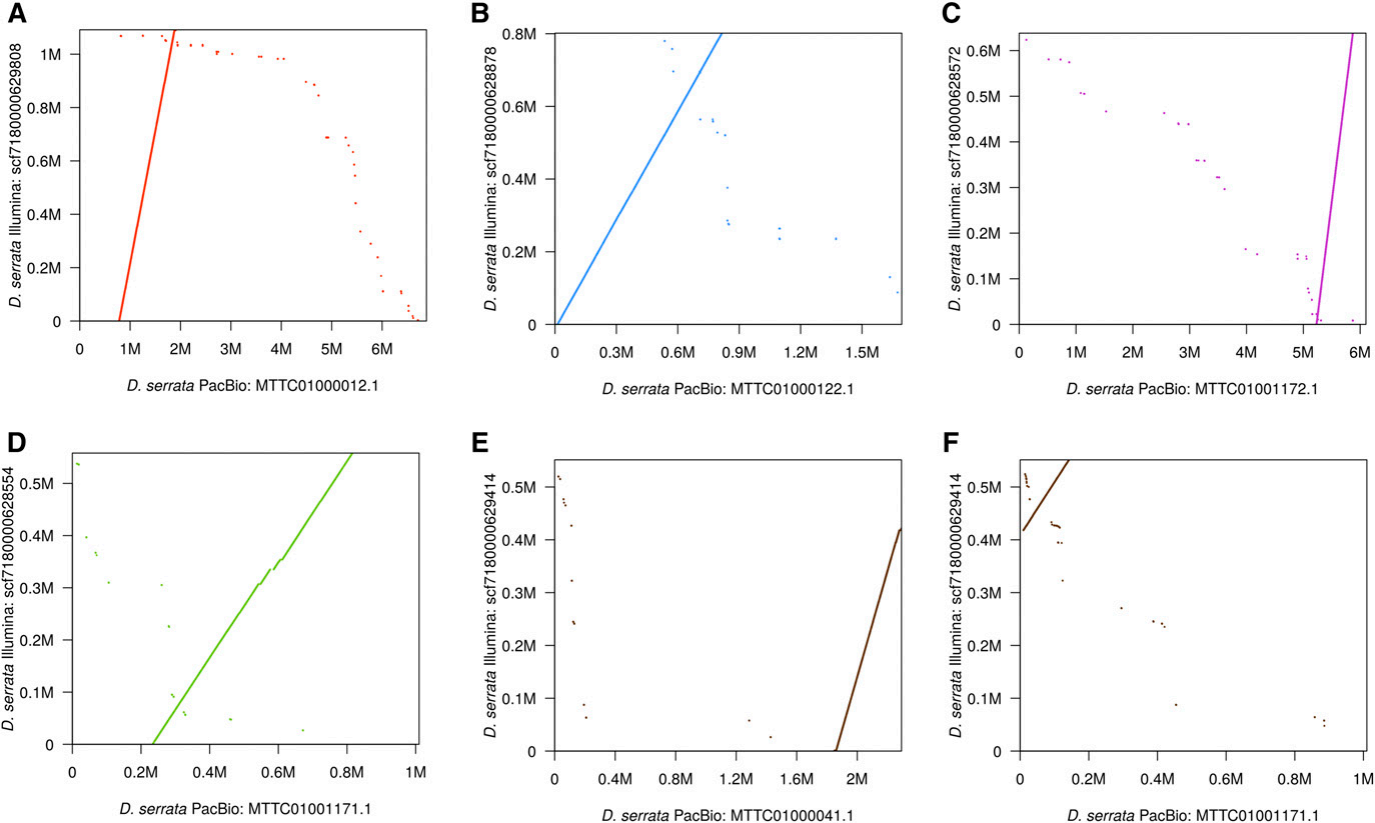
Alignments between the five longest scaffolds from our *D. serrata* assembly and orthologous contigs from a PacBio *D. serrata* assembly are highly collinear.Each dotplot shows the alignment of a scaffold from our Illumina *D. serrata* assembly (strain 14028-0681.02) to the orthologous contig from the previously published PacBio *D. serrata* assembly (strain Fors4) (Allen *et al.* 2017). Pairwise alignments were generated by LASTZ (Harris 2007). Parts A) through D) show alignments for different scaffolds. Parts E) and F) show the alignment of the same scaffold to different contigs. Alignments are shown in decreasing order of scaffold length.

To look for large-scale misassemblies, we aligned the five longest scaffolds (up to 1 Mb) from our *D. serrata* assembly (strain 14028-0681.02) to orthologous contigs in the previously published - and far more contiguous - PacBio *D. serrata* assembly (strain Fors4) (Allen *et al.* 2017). Absent large-scale misassemblies (*e.g.*, translocations, relocations, and inversions), our scaffolds should generally align end-to-end within the longer PacBio contigs, with only relatively small insertions or deletions. Dotplots for pairwise alignments are shown in Figure 4. The first four alignments are highly collinear, with our scaffolds aligning end-to-end with only relatively small insertions / deletions. The fifth alignment is also highly collinear, but our scaffold (scf7180000629414) aligns across the ends of two PacBio contigs. The dotplot pattern also suggests the presence of inverted repeats in the vicinity of the breakpoint between contigs. To determine if this represents a potential misassembly, we next aligned scf7180000629414 to the orthologous scaffold in the previously published *D. kikkawai* assembly (Chen *et al.* 2014) (Figure S2). The alignment is once again highly collinear, but this time, our entire scaffold aligns end-to-end within the longer *D. kikkawai* scaffold. Unless the *D. kikkawai* scaffold is similarly misassembled, this indicates the overall structure of our scaffold is correct. However, the fact that scf7180000629414 spans a breakpoint in a PacBio assembly suggests either the repeat structure at this locus is more complicated in strain Fors4 than strain 14028-0681.02, or our scaffold contains a local repeat-induced misassembly.

All draft genomes contain misassemblies, and ours are no different. While the above analysis generated reassuring results, it does not preclude the presence of other misassemblies (*e.g.*, collapsed repeats, small inversions, or tandem alleles) within these scaffolds. We used REAPR (Hunt *et al.* 2013) and Pilon (Walker *et al.* 2014) in our post-assembly pipeline to identify and correct as many errors as possible. While these programs work best with large-insert libraries (which we didn’t have), they nevertheless made significant improvements. We also “phased” our assemblies so that at each locus, the assembly represents the majority haplotype, within the limits of a small-insert library.

## Conclusions

We described the creation of a comparative genomic resource consisting of 23 genomes from the *Drosophila montium* species group, a large group of closely related species. Genomes for 22 of these species were presented here for the first time.

To make this endeavor financially feasible, we sequenced a single, small-insert library for each species. The absence of long-distance information made the assemblies especially sensitive to repeats and high levels of heterozygosity. As a result, many of the assemblies are fragmented, and the scaffold NG50s vary widely based on genome / sample characteristics. The total scaffold length of most assemblies is also significantly shorter than the estimated genome sizes.

However, just because most assemblies are fragmented, does not mean they are poor quality. Quite to the contrary, the BUSCO (Simão *et al.* 2015; Waterhouse *et al.* 2018) analysis showed that all assemblies, regardless of contiguity, contain at least 96% of known single-copy Dipteran genes (n = 2,799). Similarly, by aligning our assemblies to the *D. melanogaster* genome and remapping coordinates for a large set of enhancers (n = 3,457) (Kvon *et al.* 2014), we showed that each *montium* assembly contains orthologs for at least 91% of *D. melanogaster* enhancers. (This same approach can be used for any annotated feature in the *D. melanogaster* genome.) Importantly, the genic and enhancer contents of our assemblies are comparable to that of far more contiguous *Drosophila* assemblies. Finally, the alignment of our *D. serrata* assembly to a previously published PacBio *D. serrata* assembly (Allen *et al.* 2017) showed that our longest scaffolds (up to 1 Mb) are free of large-scale misassemblies.

While all of our assemblies are complete enough to study genes and enhancers, if other researchers are interested in repeat structure, any *montium* assembly can be improved on an as-needed basis. By pairing our short-read data (all of which is publicly available) with mate-pair libraries or PacBio long-reads, they can easily generate vastly more contiguous assemblies that include most repeat copies.

Going forward, our genome assemblies will be a valuable resource that can be used to further resolve the *montium* group phylogeny; study the evolution of protein-coding genes and enhancers; and determine the genetic basis of ecological and behavioral adaptations.
